# *De novo* design of molecular wires with optimal properties for solar energy conversion

**DOI:** 10.1186/1758-2946-3-S1-O14

**Published:** 2011-04-19

**Authors:** Noel M O’Boyle, Casey M Campbell, Geoffrey R Hutchison

**Affiliations:** 1 Analytical and Biological Chemistry Research Facility, University College Cork, Cork, Ireland; 2 Department of Chemistry, University of Pittsburgh, 219 Parkman Avenue, Pittsburgh, Pennsylvania 15260, USA

## 

The area of organic photovoltaic materials has elicited great interest in both the scientific and technological communities due to its potential to deliver cheap and highly efficient solar cells [1]. To date, however, such so-called molecular wires have typically yielded energy conversion efficiencies of only ~5-6% despite a theoretical maximum of 13% [2].

We present an approach that uses a genetic algorithm to search the space of synthetically accessible molecular wires for those with optimal electronic structures. This approach combines both cheminformatics (SMILES to 3D using OpenBabel) and computational chemistry (semi-empirical calculations using Gaussian09). Using this method, we have found hundreds of candidates with predicted efficiencies over 8% including many with efficiencies over 10%.
